# Vascular Endothelial Growth Factor, from Basic Research to Clinical Applications

**DOI:** 10.3390/ijms19123750

**Published:** 2018-11-26

**Authors:** Kurt Ballmer-Hofer

**Affiliations:** Biomolecular Research, Paul Scherrer Institute, 5232 Villigen, Switzerland; kurt.ballmer-hofer@unibas.ch; Tel.: +41-61-6833905; Biocenter of the University of Basel, 4056 Basel, Switzerland

Judah Folkman’s landmark discovery in the 1970s showing that tumors, growing beyond a few millimeters in diameter, depend on de novo vascularization triggered by specific growth factors released by tumor cells encountering hypoxia [1,2]. This finding led to the subsequent discovery of Vascular Endothelial Growth Factor (VEGF), also called Vascular Permeability Factor (VPF) by several laboratories [3,4,5,6]. It became clear soon after its discovery that VEGF/VPF was a member of a family of dimeric cystine crosslinked polypeptide growth factors encoded by several genes giving rise to VEGF-A, -C, -D (summarized in Reference [7]) and Placenta Growth Factor (PlGF) [8]. Structurally virtually identical homologs, collectively called VEGF-E, encoded by pox viruses of the Orf family [9,10,11] and VEGF-F, isolated from snake venoms [12,13,14,15], were subsequently isolated. An additional family member, VEGF-B, isolated more recently as a VEGFR-1 specific ligand, seemed not to display angiogenic activity but is essential for regulating fatty acid metabolism [16]. VEGF related proteins also exist in arthropods, where they regulate hemocyte development. In Drosophila, a single VEGF-like growth factor accomplishes the tasks performed by Platelet Derived Growth Factor (PDGF) and VEGF in higher organisms [17].

Besides their role in blood and lymph vessel development and homeostasis, VEGF family proteins play critical roles in the neural system, in bone development, in the hematopoietic system, and in mammalian reproductive organs. VEGF polypeptides exert their functions upon binding to type V receptor tyrosine kinases, VEGFR-1 (Flt-1), VEGFR-2 (KDR/Flk-1), and VEGFR-3 (Flt-4) [18,19,20,21]. VEGF receptors consist of seven extracellular immunoglobulin-homology domains, a transmembrane domain, and regulatory juxtamembrane domains. The intracellular tyrosine kinase domain is interrupted by a short peptide, the kinase insert domain, which is both a regulatory and a signaling element for the receptor. The carboxyterminal sequence and the kinase insert domain carry several tyrosine residues, which recruit downstream signaling molecules upon phosphorylation; they represent the actively signaling module of VEGFRs. The functional role of VEGFs and their receptors in mammalian development and in the maintenance of organ homeostasis has been the subject of intense basic biological research and we have a comprehensive understanding of the network of cellular transduction pathways activated by VEGF receptors (reviewed, e.g., in References [22,23]). The insights gained by studying VEGFR signaling led to clinical applications, e.g., in cancer therapy and in the treatment of eye diseases such as macular degeneration or diabetic retinopathy.

In this special issue of the *International Journal of Molecular Sciences*, ‘Vascular Endothelial Growth Factor’, scientists involved in basic and applied pre-clinical research present their results. A diverse collection of papers adds insights into VEGF signaling and proposes new strategies for innovative clinical applications.

Several pathophysiological vessel abnormalities are the result of aberrant signaling by VEGF family proteins, e.g., due to the atypical spatiotemporal VEGF expression in diseased tissue. This initiated the development of therapeutic tools to harness the activity of excessively produced VEGF. The first drug on the market was Bevazicumab, also called Avastin, a humanized VEGF-A-specific antibody developed by Genentech based on J. Folkman’s earlier work (reviewed in Reference [24]). This antibody clears excessive VEGF-A from tumor tissue thereby harnessing tumor growth by ablating the tumor vasculature and/or allowing the improved delivery of chemotherapeutics via ‘normalized vessels’ to tumors. Drugs blocking VEGF signaling are also successfully used in several eye diseases. Ranibizumab, an antibody related to Bevacizumab, and Aflibercept, a recombinant protein consisting of the VEGF binding epitope of VEGFR-1 and -2, are used for intra-ocular application to treat age-related macular degeneration (AMD) [25] and neoangiogenic and non-proliferative diabetic retinopathy (reviewed in References [26,27]).

In this special issue, Yi Chong Teo et al. review the successful application of anti-VEGF therapy in polypoidal choroidal neovascularization (PCV), an ocular disease related to AMD. PCV is clinically characterized by the presence of polypoidal lesions in the eye due to aberrant vascularization resulting from local overexpression of VEGF. Preliminary studies reported the stabilization of vision in PCV patients following treatment with Bevacizumab or Ranibizumab. The authors discuss the application of anti-VEGF monotherapy, therapeutic modalities such as photodynamic therapy, and the combination of these therapies.

Specific VEGF isoforms arising from alternative splicing of exon 8 discovered earlier [28] seem to play a role in several lung pathologies, such as fibrotic lung disease and rheumatoid autoimmune disease as discussed by Shaney L. Barratt et al. The balance between the fully agonistic exon 8a VEGF-A isoform, which promotes endothelial cell growth, and the partially agonistic exon 8b variant, that counteracts VEGF-A stimulated growth, may be one of the decisive factors in the development of fibrotic lung pathologies. The molecular pharmacology of exon 8 splice variants is also in-depth reviewed by Chloe J. Peach et al. The authors use sophisticated bioluminescence technology to compare exon 8a and exon 8b VEGF binding to live cells. Based on their binding and biochemical data, they propose that the ‘pluridimensional efficacy’ of VEGF signaling may display a range of signaling outputs depending on the level of receptor expression on a cell, the cell background, and the spatiotemporal activation of signaling modules by distinct ligand variants. VEGF signaling is also discussed by Akio Shimizu et al., reviewing the involvement of Rho family small G proteins such as Rho and Rap in VEGF-mediated angiogenesis, which depends on endothelial cell proliferation and migration. The authors propose that the Rho/Rap pathway may be relevant in tumor cell invasion through neuropilin-mediated GDP/GTP exchange of Rho family G proteins. The role of oxygen signaling, which regulates VEGF expression in all vascularized tissues in the developing eye is discussed by Kay D. Beharry et al. The authors present data suggesting that curtailing oxygen level variation in neonates must have a high priority to prevent severe retinopathy.

Khaled R. Alkharsah reviews the interplay between viruses and VEGF upregulation in lesions caused by specific oncoviruses, but also in general viral pathogenesis. This work shows that many viruses initiate the release of VEGF family proteins that are angiogenic and may pave the way to design novel anti-angiogenic therapeutics. Several studies found a correlation between the severity of nasopharyngeal carcinoma associated with the Epstein-Barr virus infection, its metastatic progression, and the levels of VEGF in circulation in saliva or in the tumor itself. Other viruses, such as Kaposi’s Sarcoma Herpesvirus, or Hepatitis C or B viruses, are angiogenic due to their capacity to indirectly upregulate VEGF expression. Lastly, Herpes Simplex Virus-1 or Dengue Virus upregulate VEGF expression, thereby promoting the formation of new blood and lymphatic vessels. That Orf viruses of the Parapox genus encode a particular subtype of VEGF, VEGF-E, driving angiogenesis through the activation of VEGFR-2, was already mentioned above.

Samuel J. Geiseler and Cecilie Morland reviewed the role of VEGF in neuroprotection and neurogenesis, e.g., in the recovery from stroke. The role of VEGF-A in protective mechanisms, by promoting neo-angiogenesis, and harmful effects, by causing vascular leakage and edema, in stroke faces scientists and clinicians with a dilemma. In addition, *Joon W. Shim and Joseph R. Madsen* present an overview of VEGF signaling in neural disorders and discuss the general role of VEGF in vessel homeostasis and architecture in neural tissue. Both papers advocate the idea to use VEGF-A as a potential therapeutic target in neural disorders such as ischemic stroke.

Silvia Dragoni and Patric Turowski review polarized VEGF signaling at vascular blood-neural barriers, a recent surprising finding establishing the mechanism of spatial signal processing. Cell polarity arises through asymmetric distribution of cellular components between two poles of a cell and plays a key role in intracellular transport, cell division, differentiation, cell movement, and morphogenesis. The authors describe the different responses to VEGF triggered by luminal or abluminal stimulation of vessels, which result from discrete expression of VEGFR-1 and/or -2 on either side of the blood vessels. The signal specificity of VEGF pathways is also discussed by Cristina M. Failla et al., presenting data elucidating the role of soluble truncated VEGFR-1 isoforms in positive and negative regulation of angiogenic signaling by VEGF. They show that distinct extracellular subdomains of soluble VEGFR-1 interact with integrins or glycosphingolipids expressed on the surface of endothelial cells supporting either positive or negative growth signals. Such soluble VEGFR-1 isoforms play a critical role in the development of several human pathologies. The development of innovative tools to harness VEGF signaling for basic and applied research is still welcome. Dragana Avramovic et al. present data showing that targeting an allosteric site in the extracellular domain of VEGFR-2 with recombinant antibodies is a promising approach to inhibit VEGF signaling. These antibodies were obtained using classical phage display technology and access a specific regulatory subdomain of the extracellular domain of VEGFR-2. These antibodies promote the clearance of the receptor from the cell surface via non-productive internalization and thus block signaling.

Taken together, the collection of articles published in this special issue documents that angiogenic signaling by VEGF is still a hot issue in basic biological research. Working on VEGF holds promise for unique and surprising insights into the signaling machinery driven by VEGF ligands, while the papers summarizing applied research give us a preview on future clinical applications, particularly in ophthalmology.
